# A genome-wide scan for signatures of selection in Azeri and Khuzestani buffalo breeds

**DOI:** 10.1186/s12864-018-4759-x

**Published:** 2018-06-11

**Authors:** Mahdi Mokhber, Mohammad Moradi-Shahrbabak, Mostafa Sadeghi, Hossein Moradi-Shahrbabak, Alessandra Stella, Ezequiel Nicolzzi, Javad Rahmaninia, John L. Williams

**Affiliations:** 1 Department of Animal Science, Faculty of Agriculture, Urmia University, 11Km Sero Road, P. O. Box: 165, Urmia, 5756151818 Iran; 2 Department of Animal Science, Faculty of Agricultural Science and Engineering, University College of Agriculture and Natural Resources (UTCAN), University of Tehran, P. O. Box: 4111, Karaj, 1417614418 Iran; 3 Parco Tecnologico Padano (PTP), Via Einstein, Cascina Codazza, 26900 Lodi, Italy; 4 Department of Animal Breeding and Genetics, Animal Science Research Institute of Iran (ASRI), Karaj, 3146618361 Iran; 5 Davies Research Centre, School of Animal and Veterinary Sciences, University of Adelaide, Roseworthy, SA 5371 Australia

**Keywords:** Population differentiation index, Genetic structure, Divergent selection

## Abstract

**Background:**

Identification of genomic regions that have been targets of selection may shed light on the genetic history of livestock populations and help to identify variation controlling commercially important phenotypes. The Azeri and Kuzestani buffalos are the most common indigenous Iranian breeds which have been subjected to divergent selection and are well adapted to completely different regions. Examining the genetic structure of these populations may identify genomic regions associated with adaptation to the different environments and production goals.

**Results:**

A set of 385 water buffalo samples from Azeri (*N* = 262) and Khuzestani (*N* = 123) breeds were genotyped using the Axiom® Buffalo Genotyping 90 K Array. The unbiased fixation index method (F_ST_) was used to detect signatures of selection. In total, 13 regions with outlier F_ST_ values (0.1%) were identified. Annotation of these regions using the UMD3.1 *Bos taurus* Genome Assembly was performed to find putative candidate genes and QTLs within the selected regions. Putative candidate genes identified include *FBXO9*, *NDFIP1*, *ACTR3*, *ARHGAP26*, *SERPINF2*, *BOLA-DRB3*, *BOLA-DQB*, *CLN8*, and *MYOM2*.

**Conclusions:**

Candidate genes identified in regions potentially under selection were associated with physiological pathways including milk production, cytoskeleton organization, growth, metabolic function, apoptosis and domestication-related changes include immune and nervous system development. The QTL identified are involved in economically important traits in buffalo related to milk composition, udder structure, somatic cell count, meat quality, and carcass and body weight.

**Electronic supplementary material:**

The online version of this article (10.1186/s12864-018-4759-x) contains supplementary material, which is available to authorized users.

## Background

The water buffalo (*Bubablus bubalis*) is an important livestock resource in many regions of the world, particularly in tropical and subtropical countries. Water buffalo produce milk, meat and are used as draught animal in developing countries [1, 2]. There are two types of domestic water buffalo: the river buffalo that originated in the Indian sub-continent and are now spread widely from India to Europe, and the swamp buffalo, that originated in N. Thailand or Laos and are the most common buffalo in Asia from India to the Philippines [2, 3]. The current world population of buffalo is about 200 million head compared with 1.49 billion cattle, 1.17 billion sheep and 1 billion goats [4]. Although, water buffalo represents only 11.8% of the world bovinae population, a large proportion of the world’s population depend on the domestic water buffalo [1, 5]. Unlike other domesticated bovids, whose populations are declining, the water buffalo population worldwide has increased constantly at a rate of 1.65% per year during the last five decades. However, the potential of buffalo has not been fully exploited. Water buffalo breeders and farmers face many challenges, such as poor reproductive efficiency, suboptimal production potential, and low rates of calf survival [6]. Improvement of these traits will support increasing buffalo production, particularly in poorer communities.

Iranian buffalo breeds originated from the Indian sub-continent and have been farmed in the Lorestan province of Iran since the ninth Century B.C. [7]. In the 1930s there were 1.5 million head of buffalo in Iran [7]. In contrast to the world trend, the number of Iranian buffaloes has dramatically decreased to ~ 204,000 head today [AGRI, 2014].

There are three main buffalo breeds in Iran: Azeri, Khuzestani and Mezandrani, with 119,000, 81,000 and 4000 individuals, respectively. These three breeds are reared in three different geographical areas of the country: the Azeri is widespread in the north-west and north of Iran, the Khuzestani is focused in the west and south-west (mainly in Khuzestan province), and the Mazandrani in the north of the country, mainly in the Mazandran province. These breeds experience dramatically different climatic conditions: in the northwest, Azeri buffalo are exposed to cold, sub-zero winters with heavy snowfall and hot, dry summers a with temperatures reaching 35 °C. The climate of Khuzestan in the South-west is generally very hot and occasionally humid with summertime temperatures routinely exceeding 45 °C degrees while in the winter, it can drop below freezing.

The buffalo farming system in Iran is based on smallholders (99%) and the median herd size is five animals, with a small number of herds of between 20 and 50 buffaloes and only a very few herds with 200 buffaloes (AGRI, 2010). Farming systems differ between breeds. Khuzestan buffaloes are raised outdoors throughout the year, while Azeri and Mazandrani breeds are housed in autumn and winter seasons [7]. Water buffalo provide about 239 thousand tons (2.8% of Iran's total milk production) and 24.7 thousand tons (2.5%) of meat [AGRI, 2010]. Average milk production of Iranian buffaloes in a 202-day lactation ranges from, 1141Kg in W. Azarbaijan, to 2017 Kg in Khuzestan [8]. Azeri and Khuzestani are different in milk production, body size and weight. Milk production in 210-day lactation period was 1865 and 1200 kg for Khuzestani and Azeri, respectively. Height-at-wither is 133 cm and body weight is varied 400 to 600 kg for Azeri adult female. Height-at-wither is 141 cm and body weight is 600 kg for Khuzestani adult female [7].

In recent years, the development of high-density SNP platforms has boosted genomics research in many livestock species [9]. A de novo assembly of the water buffalo genome from mixed Illumina and 454 data, and an Affymetrix panel of SNP markers has been created for water buffalo [10]. These new buffalo genomic resources open an unprecedented range of research possibilities for this species: from genome wide association studies to identifying genomic regions controlling target traits to genome assisted genetic selection [11–14].

Identifying signatures of recent selection in domesticated animals could provide information on the genomic response to domestication, climatic adaptation and selection for production traits [15]. This information may assist the design of more efficient selection schemes [16]. During the domestication process, livestock have been selected for desired morphological characteristics, physiology, increased yield, behavior and adaptation to particular environments [17, 18].

Genetic selection changes the frequency of beneficial variants and neutral variations in neighboring regions, leaving patterns in genome that can be distinguished between the population [19]. These patterns, referred to as signatures of selection, are detectable in genomic datasets as (i) changes in the ratio of non-synonymous to synonymous variations in the open reading frames (ii) a deficiency in heterozygosity compared with the rest of the genome, (iii) deviation in the Site Frequency Spectrum (SFS), (iv) differences in the allele frequencies among populations, (v) LD persistency, and (vi) unusual long-range haplotypes [20, 21]. Several statistical methods have been developed to identify selection signatures in genomic data [22–33]. To date, several studies have identified loci and genomic regions subject to positive selection in different domestic animals [18, 27, 34–36]. Once identified, these signals of selection can be used to search for the genes involved in adaptation or which are under selection [18].

The study presented here used 90,000 SNP genotypes for Azeri and Khuzestani buffalo breeds to search for selection signatures and explore putative candidate genes under the selection signatures identified.

## Results

**Fig. 1:**
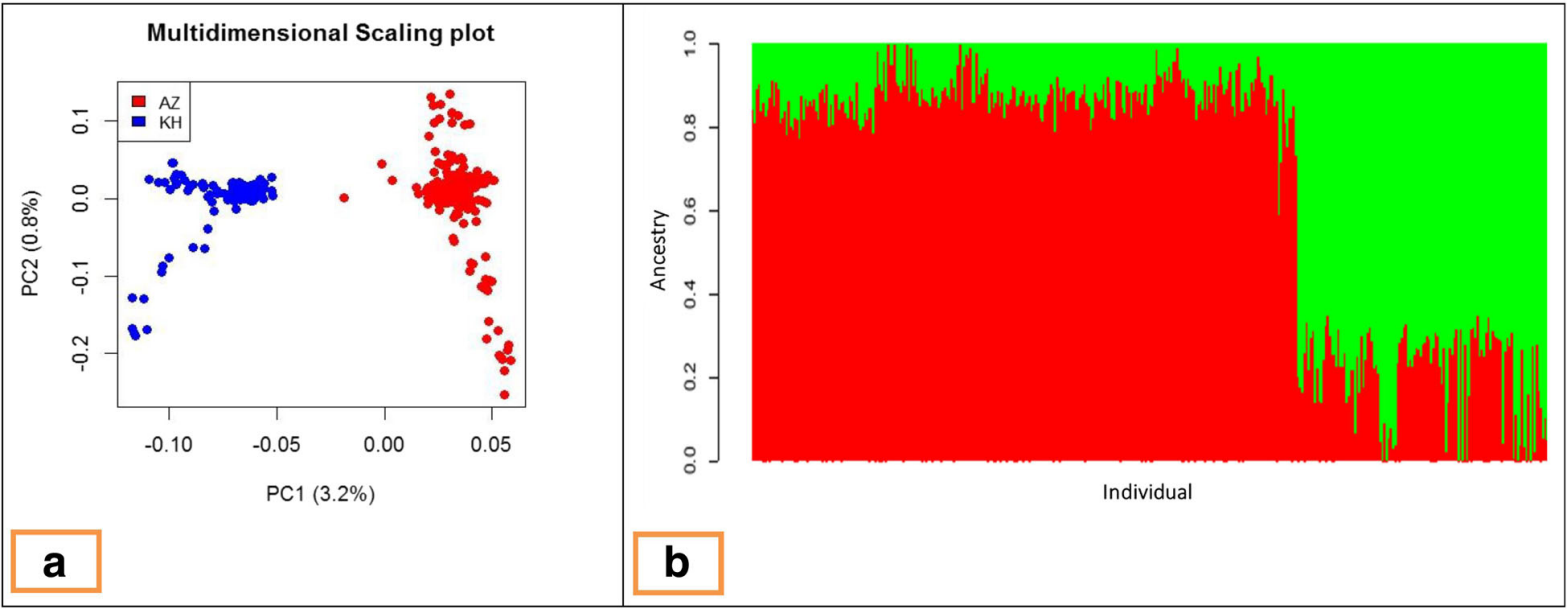
Principal components and population structure diagrams of studied buffalo populations. (**a**) Principal components diagram provided based on the genomic kinship coefficients between individuals. The first two principal components (PC) and the variance explained by each component are shown on the corresponding axis. (**b**) Population structure of studied buffalo populations provided by Admixture software with k = 2

Least square means and standard deviations of some morphometric traits in Iranian Azari and Khuzestani water buffalo breeds are shown in Additional file 1. For the SNPs analyzed in this study, the average MAF for Azeri and Khuzestani buffalo breeds were 0.317 (SD = 0.118) and 0.308 (SD = 0.122), respectively. The Azeri and Kuzestani buffalo breeds are genetically distinct, as seen by the principal components analysis [37]. Assuming two ancestral populations the structure plots show that the two breeds are distinct, but with a moderate levels of admixture (Fig. 1). The relationships among the studied animals were revealed by relationship matrix and heat map plot (see Additional files 2 and 3).

**Fig. 2:**
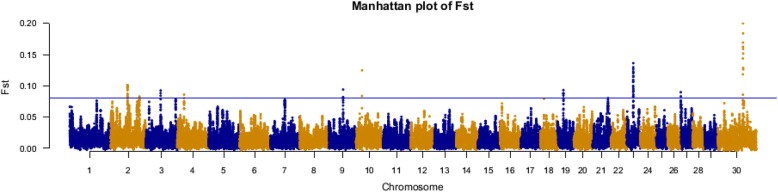
Genome-wide distribution of pairwise unbiased F_ST_ (Theta) between Azari and Khuzestani buffalo breeds. Overlapping windows of 300 kb across the genome were used to identify putative signatures of selection. The threshold determined based on the 0.1% of the empirical genome-wide distribution is shown by the blue line

Averaged Weir&Cockerham’s unbiased F_ST_ values obtained for sliding windows of 500 kb across the genome gave an average of unbiased between population F_ST_ of 0.0178 (SD = 0.027), as shown in Fig. 2. Evidence of selection was found in 13 regions which contained 0.1% of windows with the highest F_ST_ values over 0.1 included in 65 significant windows. These regions were located on chromosomes 2 (65,490–66,490 and 111,415–112,415 kb), 3 (56,750–57,750 and 114,861–115,861 kb), 4(26,287–26,287 kb), 7 (55,042–56,042 kb), 9 (54,934–55,934 kb), 10 (22,554–23,554 kb), 19 (22,916–23,916 kb), 21 (59,937–60,937 kb), 23 (24,776–25,776 kb), 27 (51.5–344 kb) and X (97,516–98,516 kb). Statistics for linkage disequilibrium, including r^2^ and D’ were calculated for regions that were selected using F_ST_ as selection signature (See Additional files 4 and 5).

The iHS and XP-EHH values were determined across the genome (see Additional files 6, 7, 8 and 9). The results revealed that the selected regions using the F_ST_ method on chromosomes 2 (2 regions), 3 (2 regions), 10, 19 and 23 were also determined as selected regions by iHS and XP-EHH methods (Additional files 6, 7, 8 and 9). The haplotype base analyses was carried out to verify selected regions from F_ST_ method. Only regions determined as signature of selection from F_ST_ were used for gene and QTL detection.

The 1 Mbp genomic regions flanking the putative signature of selection regions were searched for genes that may be have been the subjects of selection. In a total, 13 Mb representing 0.45% of the genome were included and 59 genes and 18 QTLs were identified from the annotation of the cattle genome (UMD3.1 *Bos Taurus*). The results are shown in Table 1 and Additional file 10.

**Table 1:** Complete list of genomic regions and genes harboring significant SNPs identified by unbiased F_ST_ method

Chr	Location on Cattle genome	Genes	QTL	QTL-ID	QTL Reference
2	65,990,337	GPR39- ACTR3-(bta-mir-2904-3)-(bta-mir-2887-2)	fatty acid content	20,506	[66]
2	112,415,910	SGPP2- MOGAT1- ACSL3- KCNE4	–	–	–
3	57,251,956	SNORA62	Residual feed intake	5336	[67]
3	115,361,435	SH3BP4-AGAP1	–	–	–
4	26,787,114	HDAC9	Somatic cell count	1500	[68]
			Milk protein percentage	2517	[69]
7	55,541,968	NDFIP1 – ARHGAP26	Average Daily Gain	22,812	[13]
9	55,434,304	–	Rump angle	1686	[70]
			Somatic cell count	1744	[71]
10	23,054,360	–	Teat placement	4634	[72]
			Udder attachment	4633	[72]
			Subcutaneous fat	7093	[73]
			Meat percentage	7094	[73]
			Somatic cell count	2701	[74]
			Carcass weight	4546	[75]
			Body weight (mature)	10,872	[76]
			Marbling score (EBV)	10,874	[76]
19	23,416,189	RPH3AL-DOC2B-WDR81-SERPINF2-SERPINIF1-RPA1-DPH1-OVCA2-HIC1-SRR-SGSM2	Somatic cell score	6225	[77]
21	60,437,376	ERICH1-SERPINA3–6	Shear force	20,814	[14]
23	25,276,633	TMEM14A-FBXO9-(BOLA-DQB) (HLA-DQB1)-(BOLA-DRB3)-BTNL2	Milk protein yield	3631	[78]
27	274,465	CLN8-ARHGEF10-KBTBD11-MYOM2	–	–	–
X	98,016,499	MAGED2-APEX2-RRAGB-FOXR2	–	–	–

## Discussion

Among the three Iranian buffalo breeds, Azeri and Kuzestani are the most common indigenous breeds that are well adapted to different regions. The Khuzestani have high milk, meat and growth in comparison with other breeds in Iran. The Azeri and Khuzestani breeds have differences in behavior, milk production and body size and are adapted to different environments and rearing methods. In this study, the population structure of these two buffalo breeds was analyzed. Principal component analysis (PCA) of the genotype data formed two distinct clusters with no overlap between them, each containing one of the two breeds showing that the breeds are genetically distinct (Fig. 1). Further analysis of the population structures showed that there was significant admixture (Fig. 1). The mean genomic F_ST_ value across all SNPs was 0.0178, indicating low genetic differentiation (F_ST_ ranged less than 0.05) according to Wright’s classification [38].

Iranian buffalo are exposed to extreme heat stress which is known to reduce an animal’s performance in tropical, sub-tropical and arid areas [39]. Compared with other farm animals, buffaloes have poor heat tolerance capacity and are more sensitive to heat because of scarcely distributed sweat glands and dark body color [40]. The extent of heat stress depends on the individual animal’s genetics which can alter a number of physiological and behavioral responses [41]. Adaptation to heat stress requires the physiological integration of many organs and systems including endocrine, cardiorespiratory and immune system [42]. Thus, genetic variation could be selected for better adaptation [43]. Here, 59 genes and 18 QTLs were found within the regions of high F_ST._ Some of these loci may be related to environmental adaptation such as the cytosketal organization and immune function, while others affect production traits including milk production, and growth (Table 1 and Additional file 10).

Natural selection is expected to act strongly on immunity genes through disease exposure and response to stress [18]. The highly polymorphic major histocompatibility complex (MHC) has been implicated in the resistance and susceptibility to a broad range of diseases [44], differences in milk production, growth rate, reproductive performance and immune response [45]. The bovine BoLA locus is located on BTA23 between 25.3 and 25.6 Mb which was identified as a region of high F_ST_ in the comparison of the two buffalo breeds studied here (Table 1). Other genes in this region are also involved in immune responses, specifically *FBXO9*. Also, *NDFIP1* (located on BTA7 between 54.9 and 55.9 Mb) is another gene detected in a region under selection and is involved in immune response.

Candidates genes involved in cytoskeleton organization within high F_ST_ regions included *ACTR3, ARHGAP26* and *CLN8*. Genes in this category within high F_ST_ have been implicated in muscle development, including *MYOM2* [46] which has a role in protein synthesis and modification of skeletal muscle [46], and, *ARHGAP26* and *ACTR3* involved in actin filament polymerization and organization (NCBI). MOGAT1 and AC8 L3 have key role in lipid and Fatty Acyl-CoA biosynthesis. *GPR39* (located on BTA2 between 65 and 66 Mb) is involved in the control of growth hormone release [47].

Apoptotic pathways participate in growth, proliferation, development, immunity and stress responses. Genes involved in apoptosis within high F_ST_ regions included *TMEM14A* (Trans membrane protein 14A) which stabilizing mitochondrial membrane potential [48] that may be affected by heat stress. The *CLN8* [36] has a negative regulatory function in the apoptotic process. *DNAJB2* encodes a heat shock binding protein which has anti-apoptosis function and has been implicated in meat tenderness. *SERPINF2* is involved in regulation of proteolysis, which is a response to heat stress (NCBI). *SERPINF2* is involved in the *Wnt* signaling pathway which associated with apoptosis response, but is also involved in mammary gland alveolus development, possibly related with milk production traits [49].

The QTLs that were associated with regions of high F_ST_ are shown in Table 1 and are involve traits such as fatty acid content, milk protein percentage, milk protein yield, somatic cell score, teat placement, udder attachment, subcutaneous fat, meat percentage, marbling score (EBV), shear force, carcass and body weight in mature, somatic cell count and residual feed intake traits.

## Conclusions

In this study, a genomic scan was performed on two distinct Iranian buffalo breeds and was analyzed using a population differentiation index approach. A total of 13 regions with outlier F_ST_ were detected, indicating greater than genome average divergence between the two Iranian buffalo breeds in these regions where natural or breeding selection may have been acting. A total of 59 genes were identified within these regions. Many of these genes are involved with physiological pathways including milk production, cytoskeleton organization and growth, metabolic and apoptosis processes, immune function. Hence, these genes may be considered as candidates for genes under selection. However, from this large number of candidate genes and very wide range of functions it will be necessary to refine the study to identify those under selection and variants that are beneficial for production nor climate adaptation traits.

## Methods

### Animal samples and phenotype data

**Fig. 3:**
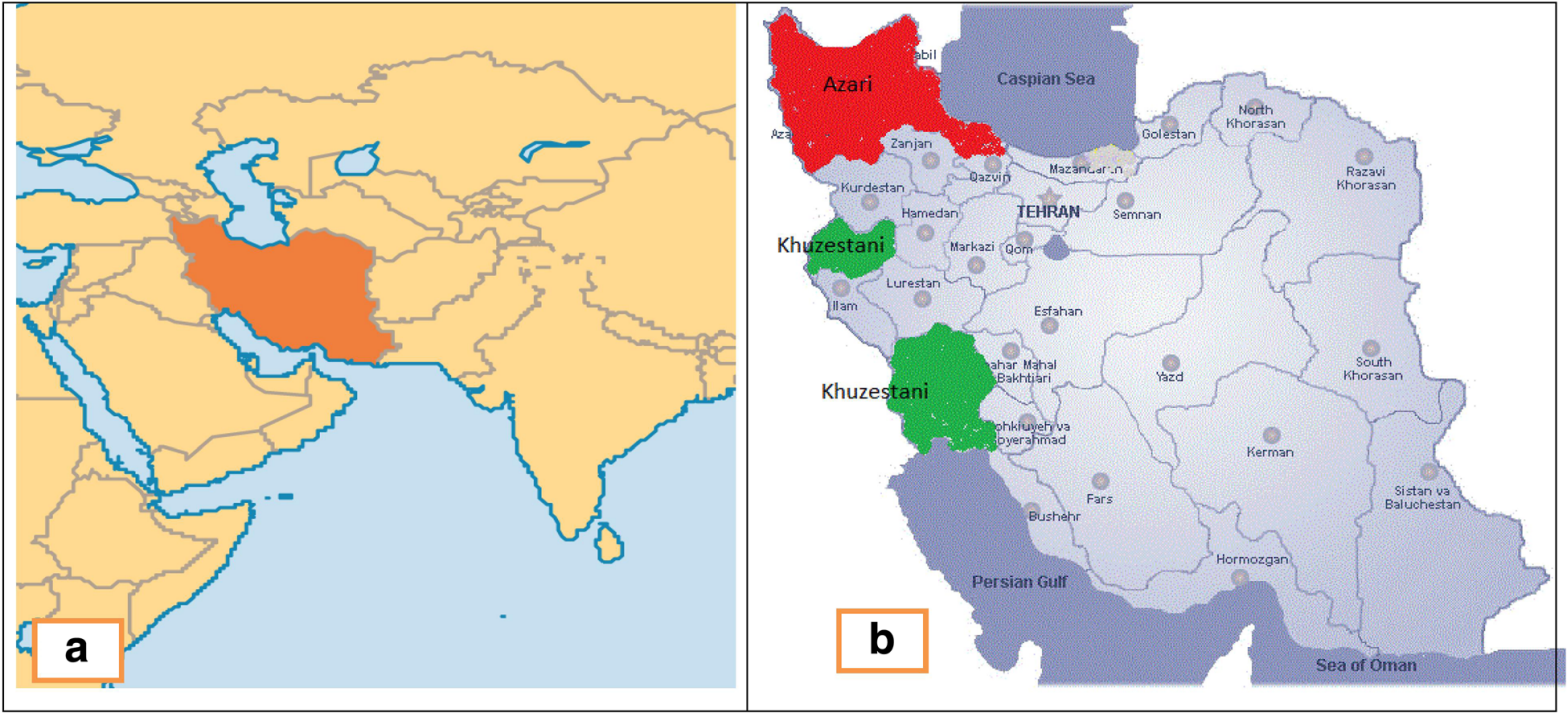
Distributions of the two Iranian Buffalo breeds used in this study. (**a**) The Iran country in Middle-East (south-west of Asia). (**b**) More than 99% of AZI and KHU buffalo breeds distribute in red and green highlighted areas, respectively. The red highlighted area (located in north and north-western part of Iran) consist of East-Azarbaijan, west-Azarbaijan, Ardebil and Gilan provinces, and the green highlighted area (west and south-western part of Iran) consist of Khuzestan and Kermanshah provinces

Blood and hair root samples were collected from 159 milk-recorded herds, including 112 herd for Azeri (AZI) breed and 47 herd for Khuzestani (KHU) breeds, respectively, which participate in the registration and recording system of the Animal Breeding Centre of Iran. Sample collection from studied animals was performed in accordance with animal ethics and approved by the Animal Use Committee in University of Tehran and Animal Breeding Center of Iran (ABCI). Fewer than 5 animals were selected from each herd. Individuals from each herd were selected based on the lowest possible pedigree-based relationship. Production records and type traits were considered to assess the diversity of the each breed. Extensive sampling was carried out to cover a large proportion of each breed. The AZI breed samples were collected from East-Azarbaijan, West-Azarbaijan, Ardebil and Gilan provinces, located in north and north-western part of Iran (37.02° – 38.78° N, 44.81° - 49.52° E), whereas the KHU breed samples were collected from Khuzestan (30.68–32.55° N, 48.02°- 48.97° E) and Kermanshah provinces (34.54°N, 45.60°E), located in the south-western part of Iran (Fig. 3). In total 510 samples were collected from which 385 were selected for genotyping. Selected animals had milk production records and type traits including height-at-withers and chest girth. The data, normally distributed in each breed, were analyzed by SAS software (SAS 2014, SAS Institute Inc., Cary, NC, USA) using the GLM procedure. The results showed that breed had significant effect on traits (*P* < 0.001) and KHU had higher height-at-withers (143.63 vs 138.73), chest girth (195.37 vs 183.89) and milk production (10.92 vs 6.98) than AZI.

### Genotyping and data quality control

Genomic DNA was extracted from blood by the modified salting out method [50] and from hair samples as described by Alberts et al. [51]. The quality and concentration of extracted DNA were assessed by visualizing on 1.2% agarose gel and spectrophotometrically based on absorbance at (260 nm /280 nm). DNA samples were diluted to 50 ng/ul for genotyping.

The set of 385 water buffalo samples from AZI (*N* = 262) and KHU (*N* = 123) breeds with milk production and type records were genotyped using the Axiom® Buffalo Genotyping 90 K Array (Affymetrix). SNP genotypes were extracted from raw data using the AffyPipe workflow [52] and applying default thresholds (dish-quality control < 0.82 and individual call rate < 0.97). Primary quality control and filtering, was carried out and genotypes exported in PLINK format. In total, 4 animals were removed because they failed the quality standards. Furthermore, 5501 (6.1%) and 9857 (11.7%) SNP were discarded because the call rate was below the threshold and low quality genotypes, respectively. A total of 73,935 SNP passed the quality control which had an average sample call rate of 99.68%. Genotype repeatability assessed from 5 replicate samples was 99.96%, demonstrating a high quality of the genotyping results. A total of 64,339 (71.6%) probes were high-quality polymorphic (PolyHighResolution class), 7924 (8.8%) showed high-quality monomorphic signals (MonoHighResolution) and 1672 (1.8%) had one homozygous genotypes class missing (NoMinorHom). The latter three classes were retained for further analyses. Therefore, the quality-edited dataset has a total of 383 animals, AZI *n* = 260 and KHU *n* = 123 individuals and 73,935 SNP genotypes.

A second QC procedure was performed breed-wise, using PLINK software [53], retaining SNPs on autosomal and X chromosomes, minor allele frequency (MAF) > 0.02%, divergence from Hardy-Weinberg Equilibrium (HWE) (*P*-value >10e-6) and SNP Call rate (CR_SNP_) > 0.95%. After this quality control, individuals with call rate (CR_IND_) below 0.95% were excluded from further analysis (Table 2 and Additional file 11). This procedure yielded 371 individuals (253 AZI and 118 KHU) and 64,866 SNPs with average distances between 2 adjacent SNPs about 40 kb based on the bovine genome UMB 3.1, which were used for further analyses (Table 2).

**Table 2:** Description of AZI and KHU buffalo breeds genotypes available for analysis before and after filtering for cryptic quality control

	AZI	KHU
Number of individuals before filtration	260	123
Number of SNPs before filtration	73,935	73,935
SNPs with unknown position on genome	19	19
SNPs with minor allele frequency (MAF)	8830	8830
SNPs out of HWE (*P*-value <10e-6)	198	124
Total genotyping rate in remaining individuals	0.9974	0.9954
SNPs with Call rate (CR_SNP_) < 0.95%	0	0
Individuals with call rate (CR_IND_) < 0.95%	2	2
Replicate individuals	2	3
Removed individuals by PCA output	3	0
Removed individuals with IBS > 0.8	0	0
Number of individuals before filtration	253	118
Number of SNPs after QC	64,866	64,866

### Population structure and F_ST_ estimation

PCA analysis based on SNPs which passed quality control, was carried out using the identical by state (IBS) matrix generated with GenABEL [54] by converting the calculated genomic kinship coefficients to squared Euclidean distances that capture the differences between individuals via classical multidimensional scaling [55]. Individuals located outside the expected breed cluster were excluded from further analysis. As the selection of individuals was based on pedigree, the identity-by-state (IBS) relationship matrix was used to remove closely related animals, as proposed by Leutenegger et al. [56]. The IBS matrix was estimated using GenABEL R package IBS function [54] and individuals with an IBS > 0.8 were removed from further analysis. Genetic structure of the population was tested using ADMIXTURE software [57]. The r^2^ statistics between adjacent SNP pairs were calculated for both of the studied populations for all marker pairs, using SnppldHD software (Sargolzaei M, University of Guelph, Canada).

The unbiased Fixation index (Theta) estimator proposed by Weir and Cockerham was calculated (Additional file 12) to detect signatures of selection [32, 58] in R (the R project website, http://www.r-project.org/). The F_ST_ outlier method was used to detect signatures of selection [59] where adjacent SNPs show outlier F_ST_ values [60]. A modified sliding window (SW) approach (referred to as a “Creeping Window”: CW) was used to scan the entire genome for evidence of selective sweeps, using a one SNP step [61]. The optimal size of the window depends on time since the occurrence of the selection sweep, as LD breaks down with time [35]. An arbitrary 300Kbp window size was chosen in this study (Additional file 13). In total, 13 regions exceeding the 0.1% threshold of the empirical outlier window-wise unbiased F_ST_ values. Overall unbiased F_ST_ was calculated using Weir and Cockerham method [32] in R as population differentiation index. To verify selected regions from unbiased F_ST_ results and appropriately identify selection signatures, two haplotype base methods integrated Haplotype Homozygosity score (iHS) [31] and Cross Population Extended Haplotype Homozygosity (XP-EHH) were applied using rehh package [62] in R. Imputation of missing data and haplotype phasing were carried out by fastPHASE software [63] for use in haplotype base analysis.

### Annotation of the outlier regions

The 13 outlier genomic regions were surveyed to find genes within 1 Mb of the outlier region peaks. In total 59 genes were extracted from the corresponding areas in UMD3.1 *Bos Taurus* Genome Assembly using Biomart. DAVID [64] was used to perform a gene ontology analysis and to identify putative biological networks including the genes found in outlier regions. Finally, the Enrichment Map Cytoscape plug-in was used to construct networks [65].

## Additional files

Additional file 1: Least-square means and standard deviations of some morphometric and body size traits in Iranian Azeri and Khuzestani water buffalo breeds. (DOCX 19 kb)

Additional file 2: Genomic matrix of studied Iranian Azeri and Khuzestani buffalo breeds. (XLS 1750 kb)

Additional file 3: HeatMap of studied Iranian Azeri and Khuzestani buffalo breeds. (PDF 235 kb)

Additional file 4: Haploview LD graph for selected regions. (PDF 904 kb)

Additional file 5: Adjecent r^2^ information. (XLS 67 kb)

Additional file 6: Genome wide distribution of iHS and XP-EHH plot. (PDF 349 kb)

Additional file 7: Genome wide distribution of iHS from Azeri buffalo breed values. (TXT 2913 kb)

Additional file 8: Genome wide distribution of iHS from Khuzestani buffalo breed values. (TXT 2864 kb)

Additional file 9: Genome wide distribution of XP-EHH from Azeri and Khuzestani buffalo breed values. (TXT 2963 kb)

Additional file 10: Genes included in selected regions (Biomart input & output). (XLS 105 kb)

Additional file 11: Map file after QC. (TXT 1809 kb)

Additional file 12: F_ST_ output file. (XLS 9242 kb)

Additional file 13: Windowed F_ST_ output file. (XLS 6816 kb)

## Abbreviations

AZI: Azeri
CR_IND_: Individuals Call Rate
CR_SNP_: SNP Call Rate
CW: Creeping Window
F_ST_: Population differentiation index
HWE: Hardy-Weinberg Equilibrium
IBS: Identical By State
KHU: Khuzestani
MAF: Minor Allele Frequency
PCA: Principal component analysis
QC: Quality Control
SW: Sliding Window
