# Supplementary material for: A genome-wide scan for signatures of selection in Azeri and Khuzestani buffalo breeds
Source: BMC Genomics. 2018 Jun 11;19:449. doi: 10.1186/s12864-018-4759-x (PMC5996463; doi:10.1186/s12864-018-4759-x)
Supplement: Supplementary file 1 — Least-square means and standard deviations of some morphometric and body size traits in Iranian Azeri and Khuzestani water buffalo breeds. (DOCX 19 kb) [file 12864_2018_4759_MOESM1_ESM.docx]

| Table 1. Least-square means and standard deviations of some morphometric and body size traits in Iranian Azari and Khuzestani water buffalo breeds | | | | | | | | |
| --- | --- | --- | --- | --- | --- | --- | --- | --- |
| Factor | Df/N | shoulder height (SH) | Chest depth (CD) | Body height (BH) | Body depth (BD) | Body length (BL) | chest circumference (CC) | Hip width (HIW) |
| Genotype | 1 | 693.32*** | 64.82ns | -- | -- | 302.40 ns | 5576.52*** | 250.48*** |
| Khuzestani | 148 | 143.63^b^ ±1.03 | 77.30±0.86 | -- | -- | 138.45±1.73 | 195.37^b^ ±2.89 | 57.29^b^±0.72 |
| Azari | 336 | 138.73^a^ ±0.35 | 75.79±0.29 | -- | -- | 135.04±0.57 | 183.89^a^ ±1.95 | 54.3^a^±0.25 |
| Province (Genotype) | 4 | 613.20*** | 196.35*** | 196** | 93.04* | 2897.63*** | 620.40 ns | 157.33*** |
| Khuzestan | 136 | 144.8^d^±0.58 | 77.28^b^±0.50 | -- | -- | 137.13^c^±1.05 | 195.37^b^ ±2.89 | 56.01^c^ ±0.43 |
| Kermanshah | 11 | 142.44^c^±2.05 | 77.2^ab^±1.71 | -- | -- | 139.8^bcd^ ±3.43 | -- | 58.57^c^ ±1.44 |
| W- Azarbayjan | 82 | 142.5^cd^±0.69 | 78.51^b^±0.58 | -- | -- | 144.59^d^ ±1.11 | 180.84^a^ ±2.63 | 55.88^c^ ±0.48 |
| Gilan | 102 | 135.5 ^a^±0.61 | 74.90^a^±0.51 | 132.26^a^±0.63 | 73.26^a^±0.5 | 128.46^a^ ±1.02 | 181.50^a^ ±2.65 | 52.52^a^ ±0.42 |
| E-Azarbayjan | 76 | 139.7^bc^±0.70 | 74.62^a^±0.58 | 135.19^b^±0.71 | 72.52^a^±0.55 | 133.80^b^ ±1.15 | 191.94^ab^±5.16 | 55.05^bc^ ±0.49 |
| Ardebil | 77 | 137.2 ^a^ ±0.69 | 75.11^a^±0.57 | 132.73^a^±0.69 | 74.69^b^±0.54 | 133.32^b^ ±1.11 | 181.29^a^±3.81 | 53.76^ab^ ±0.48 |
| parity N | 5 | 184.91*** | 303.88*** | 47.08 ns | 155.78*** | 753.50*** | 844.54 ns | 348.22*** |
| Heifer | 40 | 139.01^a^±0.96 | 72.67^a^ ±0.81 | 132.4^ab^±1.21 | 69.65^a^±0.95 | 129.87^a^ ±1.60 | 179.98^a^±6.33 | 49.59^a^±0.69 |
| First | 62 | 139.22^a^±0.94 | 75.10^b^±0.80 | 133.4^ab^±1.07 | 72.14^ab^±0.83 | 134.3^ab^ ±1.59 | 186.68^ab^±4.01 | 55.32^b^ ±0.67 |
| Second | 78 | 140.7^ab^±0.87 | 75.60^b^±0.73 | 133.5^ab^±0.89 | 74.04^bc^±0.69 | 136.05^b^±1.48 | 188.08^ab^±3.13 | 56.48^b^ ±0.61 |
| Third | 83 | 143.16^c^±0.85 | 78.41^c^±0.71 | 135.14^a^±1.46 | 75.47^c^±0.65 | 139.63^c^ ±1.42 | 193.05^ab^±3.18 | 57.73^bc^ ±0.6 |
| Fourth | 73 | 142.6^bc^±0.88 | 78.88^c^±0.74 | 133.6^ab^±1.02 | 74.27^bc^±0.77 | 140.77^c^ ±1.48 | 196.09^b^ ±3.61 | 57.99^c^ ±0.63 |
| Fifth&more | 148 | 142.4^bc^±0.74 | 78.59^c^±0.62 | 132.39^b^±0.81 | 75.38^c^±0.63 | 139.88^c^ ±1.24 | 193.91^b^ ±2.51 | 57.68^c^ ±0.52 |
| Error | | 35.77 | 24.63 | 35.59 | 36.36 | 21.67 | 455.14 | 17.35 |
|  | | | | | | | | |

Continue Table 1:

| Table 1. Least-square means and standard deviations of some morphometric and body size traits in Iranian Azari and Khuzestani water buffalo breeds | | | | | | | | | |
| --- | --- | --- | --- | --- | --- | --- | --- | --- | --- |
| Factor | Df/N | Pip width (PW) | hip to pin length (HP) | Face length (FL) | Horn length (HL) | Inside horn interspace(OHI) | Tag end horn interspace(THI) | Fore teat length (TL) | Rear teat length (TL) |
| Genotype | 1 | 0.05 ns | 96.11 ns | 14.31 ns | 517.83** | 17.78 ns | 3663.33*** | 0.2 ns | 4.57 ns |
| Khuzestani | 148 | 26.03±0.52 | 45.41±1.27 | 51.08±0.48 | 41.12^b^±1.42 | 20.01±0.66 | 39.80^b^±2.08 | 6.63±0.38 | 7.81±0.32 |
| Azari | 336 | 26.07±0.18 | 43.56±0.43 | 50.31±0.22 | 36.36^a^±0.68 | 19.16±0.30 | 27.75^a^±0.98 | 6.69±0.41 | 8.06±0.29 |
| Province (Genotype) | 4 | 397.40*** | 95.49 ns | 19.68 * | 142.68* | 48.11** | 196.42 ns | 31.40*** | 96.23*** |
| Khuzestan | 136 | 24.24^ab^±0.3 | 44.67±0.75 | 51.33^b^±0.30 | 39.32^b^±0.87 | 21.35^b^±0.45 | 44.92±1.36 | 6.63^b^±0.38 | 7.81^b^±0.32 |
| Kermanshah | 11 | 27.83^c^±1.03 | 46.15±2.54 | 50.8^ab^±0.95 | 42.92^b^±2.84 | 18.66^ab^±1.3 | 34.68±4.11 | -- |  |
| W- Azarbayjan | 82 | 29.1^cd^±0.35 | 44.70±0.85 | 51.46^b^±0.64 | 36.8^ab^±2.03 | 21.21^b^±0.88 | 28.25±2.98 | 5.05^a^±0.54 | 6.01^b^±0.41 |
| Gilan | 102 | 23.50^a^±0.30 | 42.42±0.75 | 49.48^a^±0.28 | 34.28^a^±0.86 | 18.98^a^±0.39 | 28.4±1.23 | 6.46^b^±0.57 | 8.75^c^±0.35 |
| E-Azarbayjan | 76 | 26.69^c^±0.35 | 44.48±0.86 | 50.5^ab^±0.37 | 36.1^ab^±1.01 | 18.44^a^±0.48 | 27.1±1.49 | 7.37^bc^±0.53 | 8.47^bc^±0.4 |
| Ardebil | 77 | 25.06^b^±0.35 | 42.63±0.86 | 49.84^a^±0.34 | 38.33^b^±1.11 | 18.00^a^±0.47 | 27.25±1.52 | 7.90^c^±0.67 | 9.02^c^±0.38 |
| parity N | 5 | 141.14*** | 168.02** | 44.41*** | 2032.52*** | 13.14 ns | 136.16 ns | 22.43*** | 44.21*** |
| Heifer | 40 | 22.95^a^±0.49 | 41.88^a^±1.22 | 49.05^a^±0.49 | 29.02^a^±1.43 | 19.65±0.65 | 34.37±2.01 | 4.03^a^±2.05 | 5.52^a^±1.54 |
| First | 62 | 24.99^b^±0.48 | 43.3^ab^±1.18 | 49.99^ab^±0.50 | 32.68^a^±1.48 | 19.24±0.68 | 33.86±2.10 | 5.90±0.45 | 7.32^a^±0.33 |
| Second | 78 | 26.41^c^±0.44 | 43.9^ab^±1.08 | 50.75^bc^±0.45 | 37.26^b^±1.39 | 19.18±0.61 | 31.42±1.97 | 6.93^a^±0.34 | 7.95^a^±0.33 |
| Third | 83 | 27.3^cd^±0.43 | 45.7^bc^±1.05 | 50.82^bc^±0.43 | 41.92^c^±1.28 | 19.62±0.59 | 32.77±1.88 | 6.99^a^±0.36 | 8.30^a^±0.27 |
| Fourth | 73 | 26.9^cd^±0.45 | 45.6^bc^±1.12 | 51.55^cd^±0.46 | 43.84^c^±1.41 | 19.44±0.65 | 34.64±2.06 | 8.01^b^±0.33 | 9.10^b^±0.28 |
| Fifth&more | 148 | 27.73^d^±0.37 | 46.5^bc^±0.92 | 51.99^d^±0.37 | 47.71^d^±1.08 | 20.37±0.52 | 35.58±1.64 | 8.08^b^±0.25 | 9.49^b^±0.20 |
| Error | | 8.91 | 44.38 | 7.07 | 58.58 | 13.6 | 139.44 | 4.19 | 4.69 |
| The means within the same row withat least one common letter, do not have significant difference (P>0.05).  The “ns”: no significant, “*”: (P<0.05), “**”: (P<0.01) and “***”: (P<0.05). | | | | | | | | | |
