# Supplementary material for: A genome-wide scan for signatures of selection in Azeri and Khuzestani buffalo breeds
Source: BMC Genomics. 2018 Jun 11;19:449. doi: 10.1186/s12864-018-4759-x (PMC5996463; doi:10.1186/s12864-018-4759-x)
Supplement: Supplementary file 3 — HeatMap of studied Iranian Azeri and Khuzestani buffalo breeds. (PDF 235 kb) [file 12864_2018_4759_MOESM3_ESM.pdf]

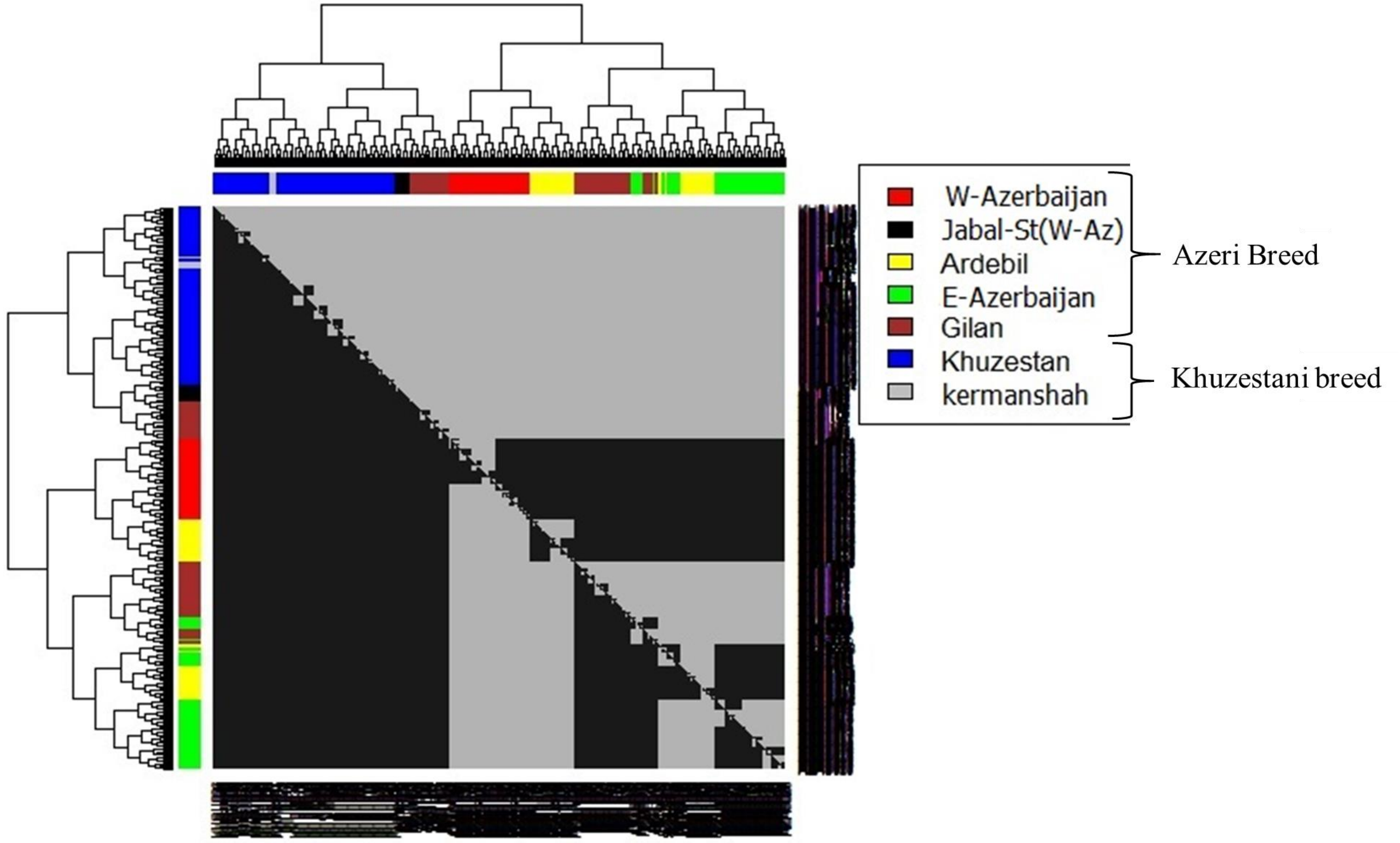

Additional File 2: Figure 1- Heatmap generated from genomic data of Azeri and Khuzestani buffalo breeds (Each province and Jabal station-related to Azeri breed in west Azerbaijan- was shown by different colors).
