# Supplementary figures and images for: A genome-wide scan for signatures of selection in Azeri and Khuzestani buffalo breeds
Source: BMC Genomics. 2018 Jun 11;19:449. doi: 10.1186/s12864-018-4759-x (PMC5996463; doi:10.1186/s12864-018-4759-x)

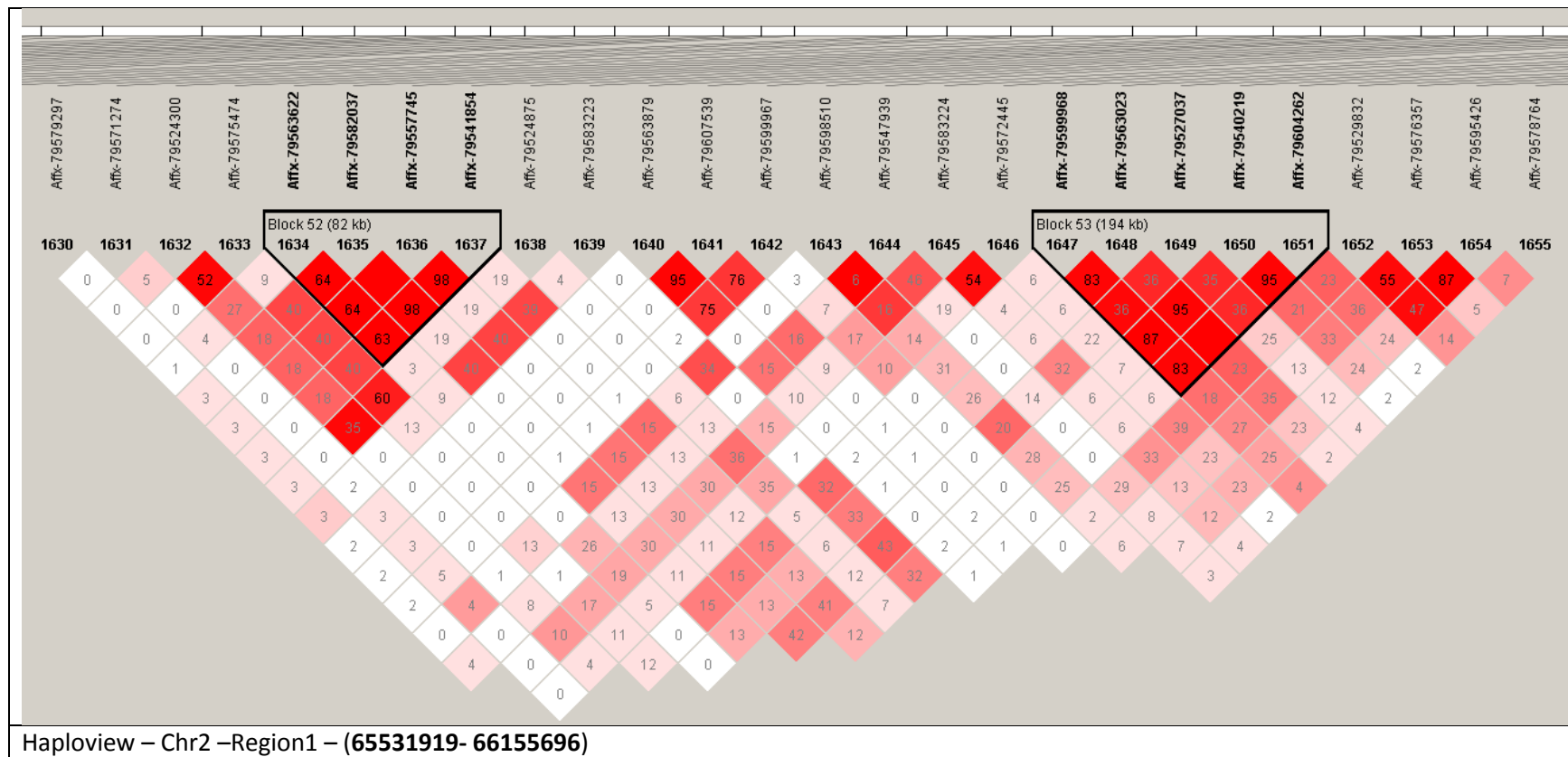

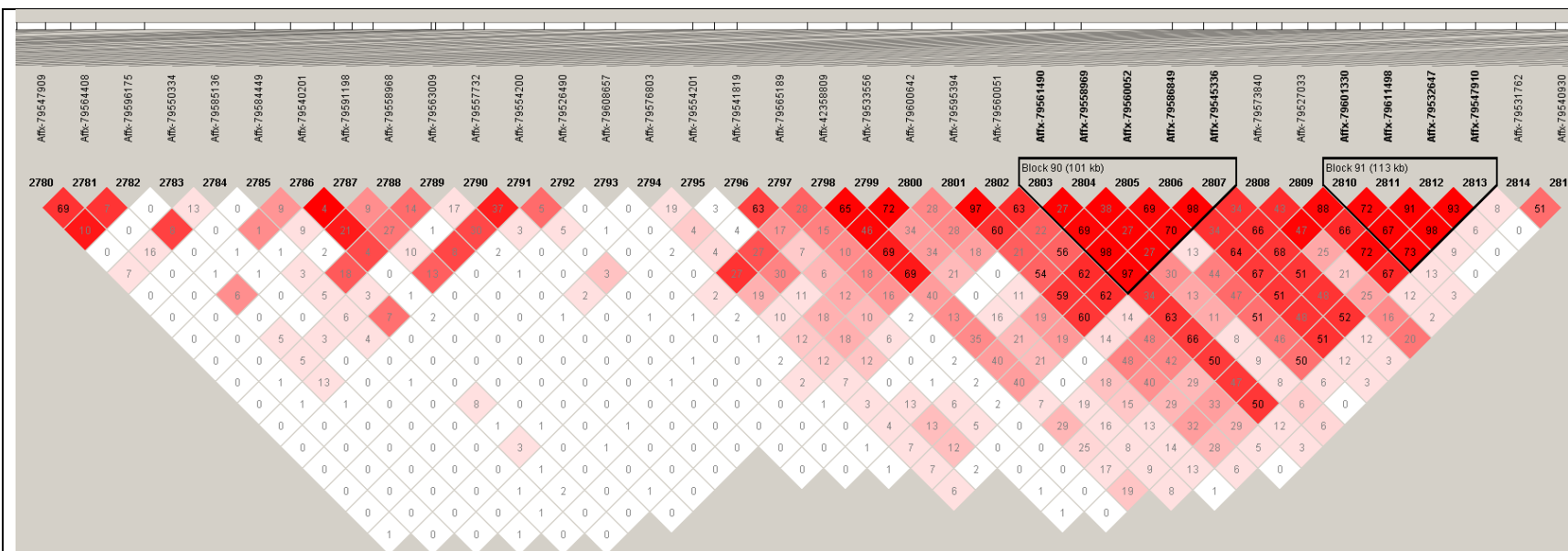

Haploview – Chr2 –Region2 – (111409740- 112505570)

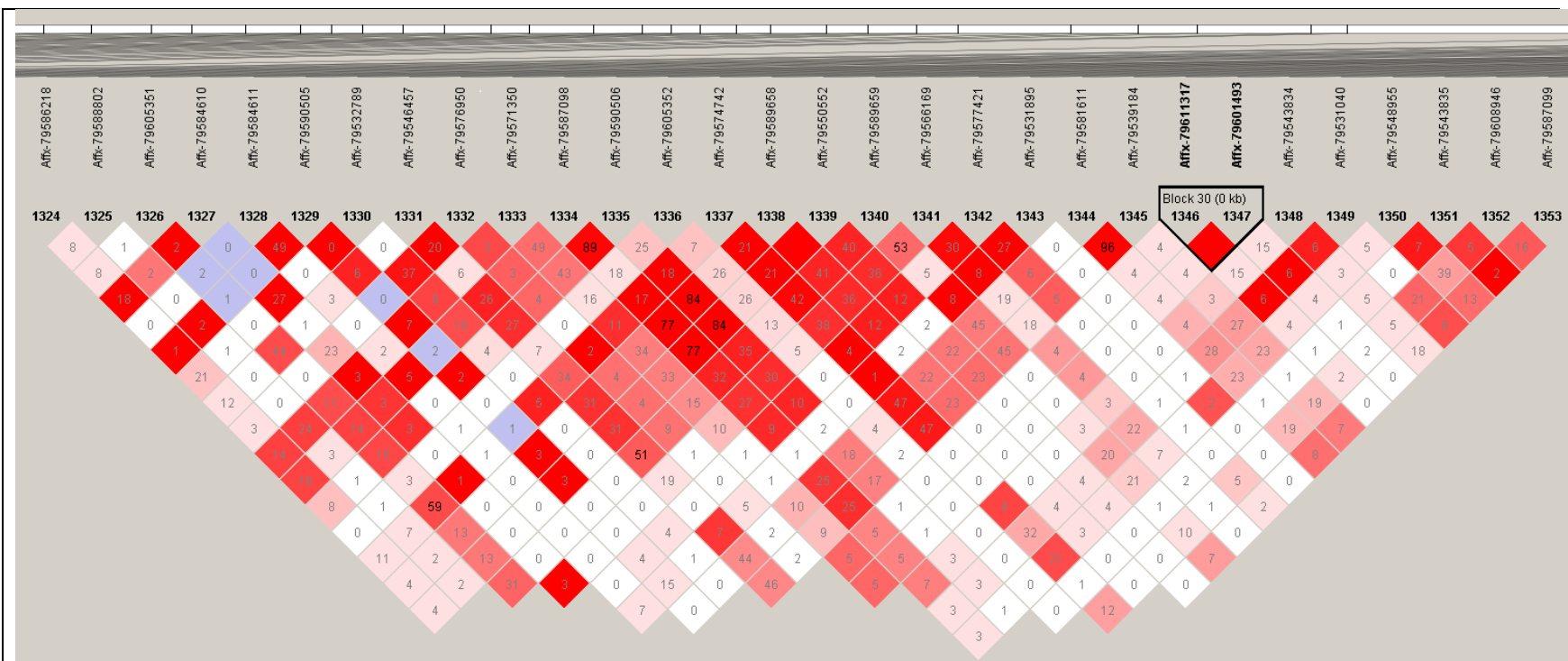

Haploview – Chr3 –Region1 – (56751956-57751956)



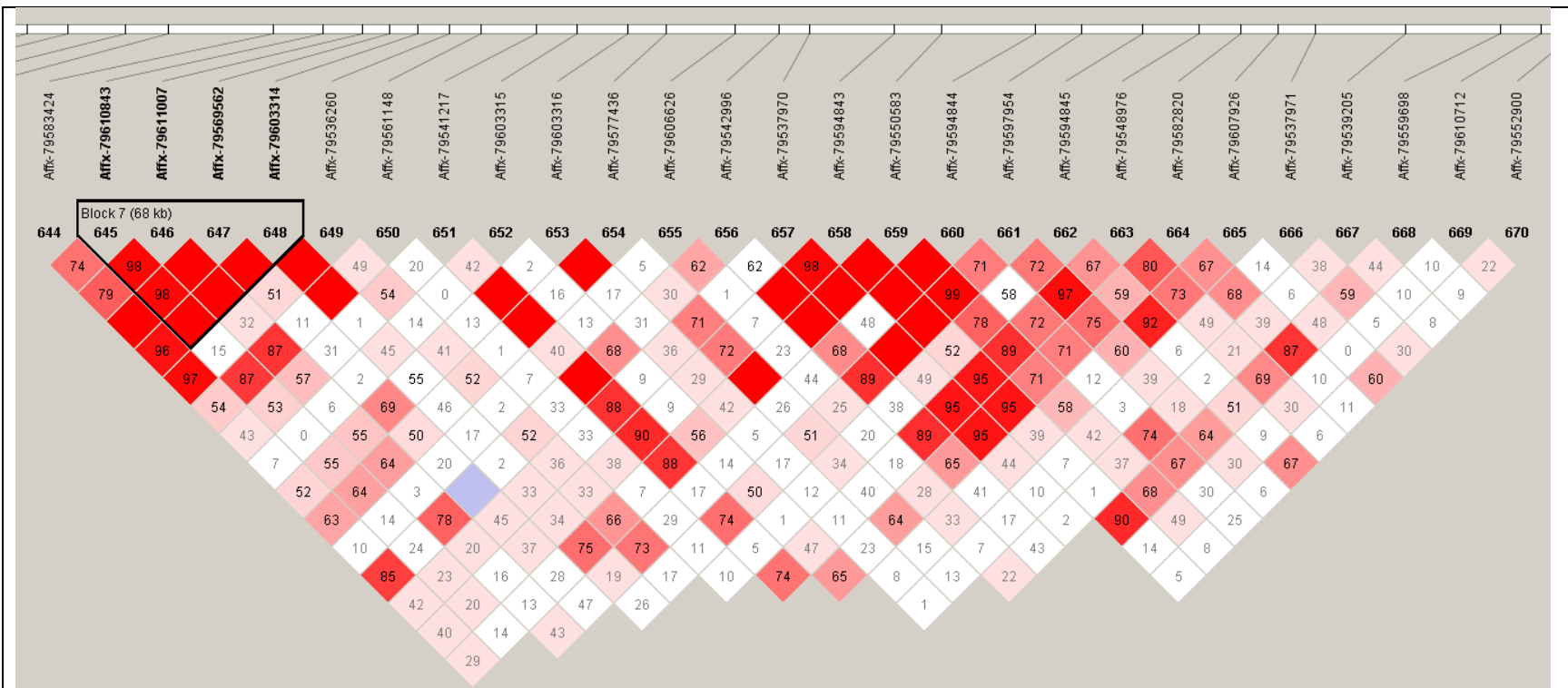

Haploview – Chr4– (26287114-27287114)

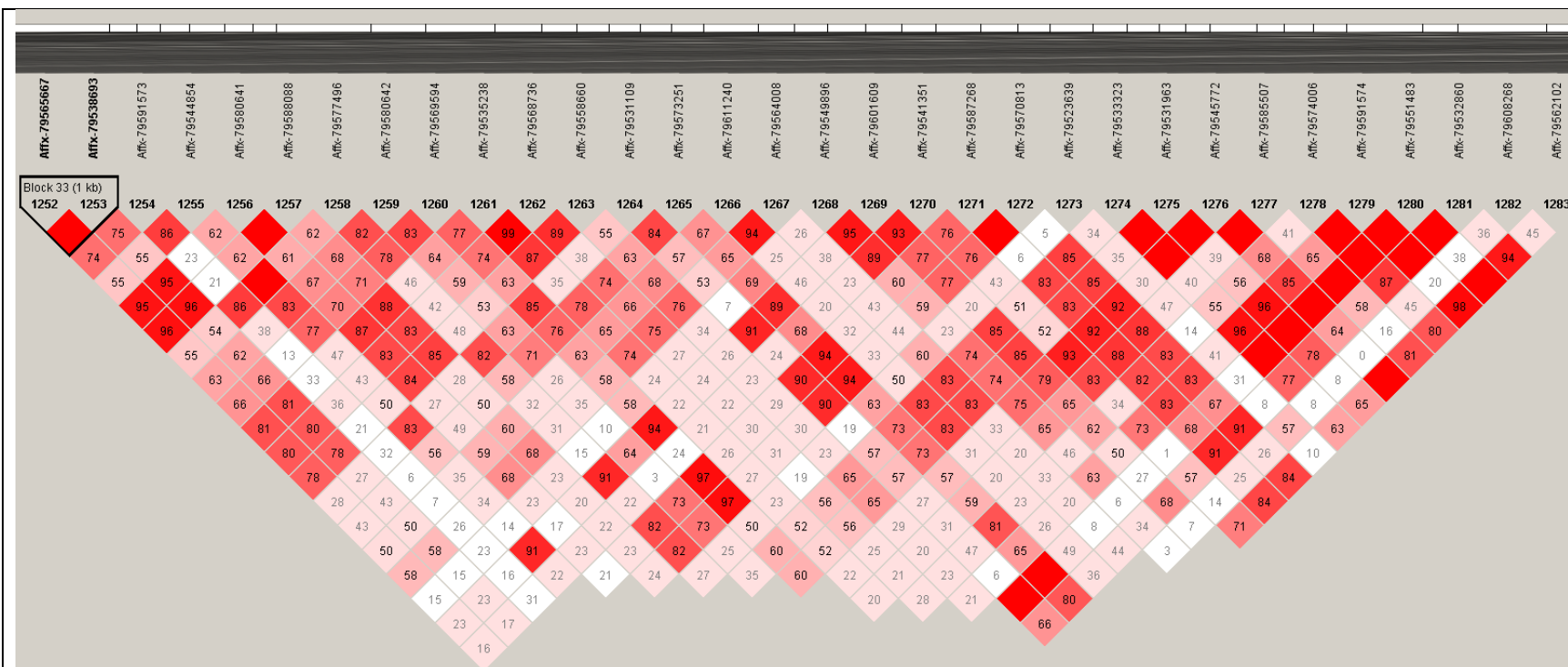

Haploview – Chr7– (55041968-56041968)

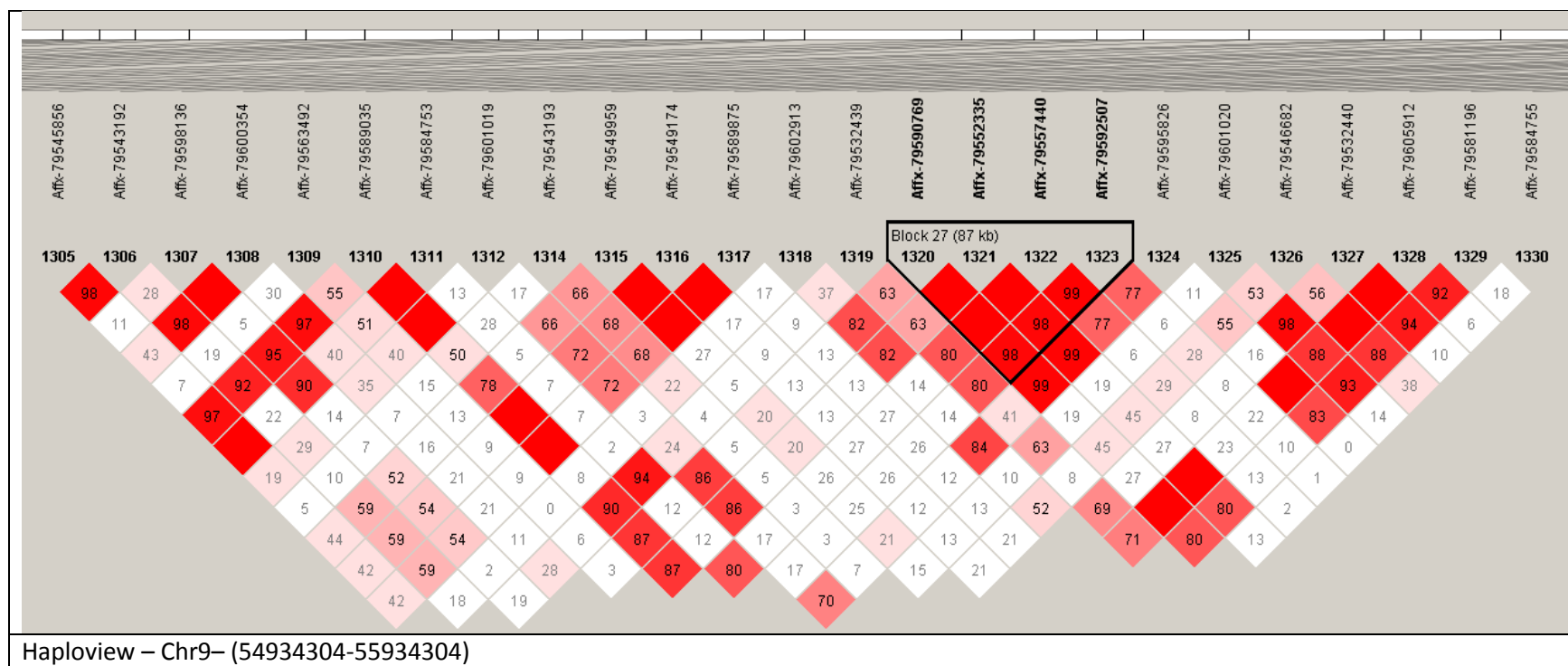

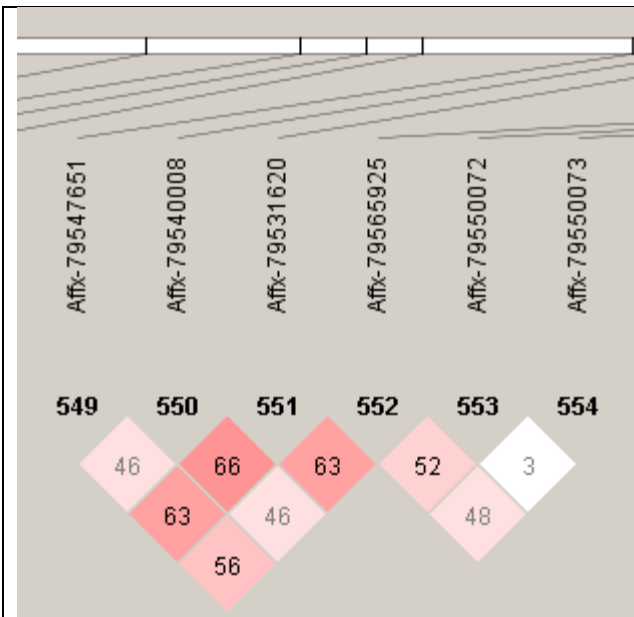

Haploview – Chr10– (22554360-23554360)

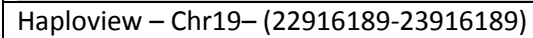

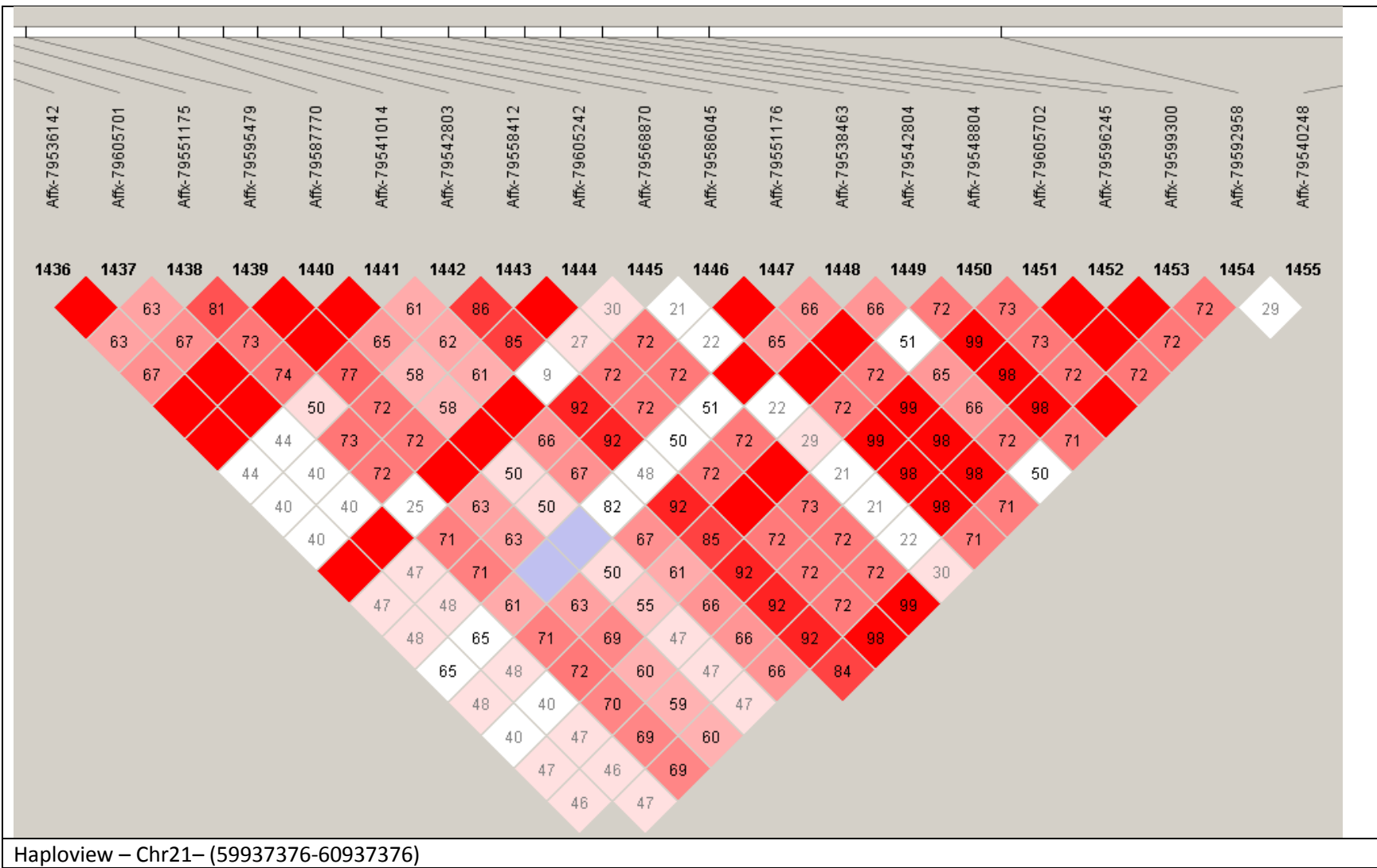

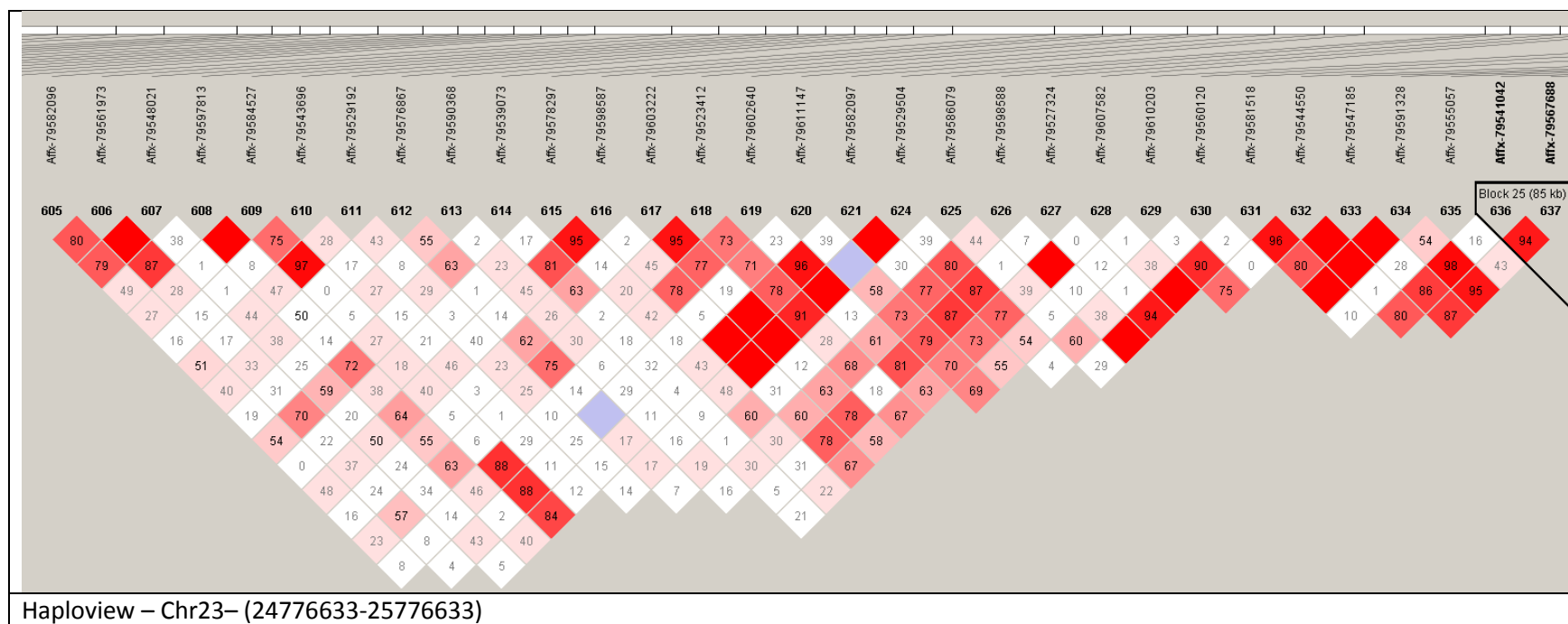

Supplement: Supplementary file 4 — Haploview LD graph for selected regions. (PDF 904 kb) [file 12864_2018_4759_MOESM4_ESM.pdf]

Pvalue AZI -KHU XP-EHH

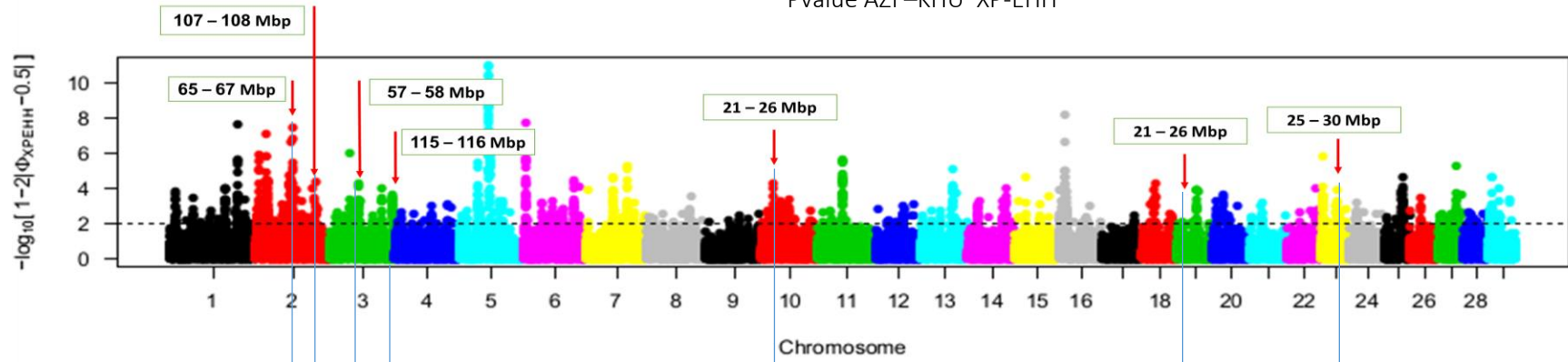

Pvalue AZI iHS

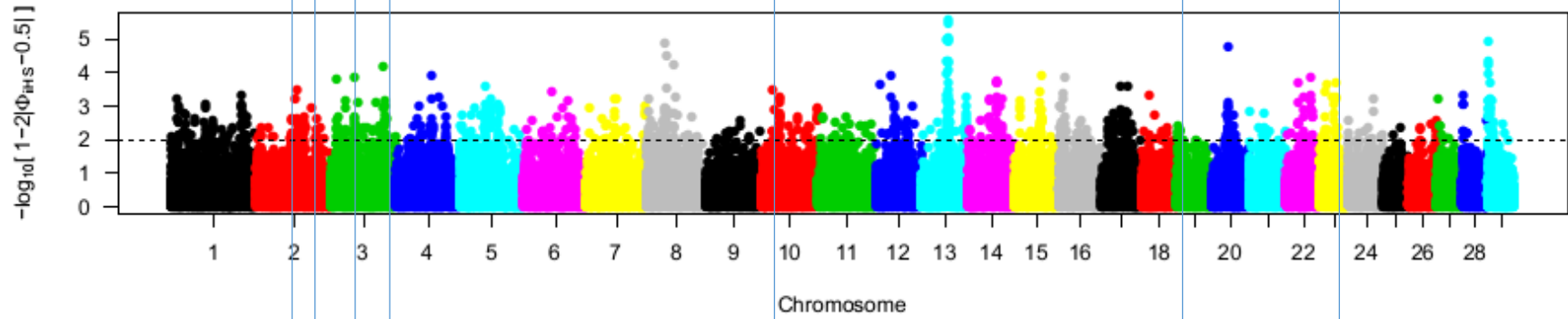

Pvalue KHU iHS

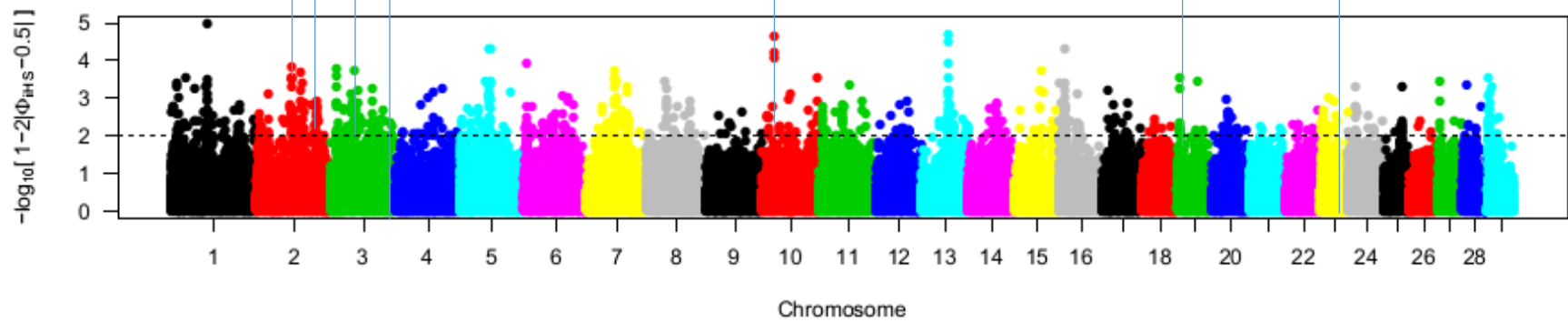

Supplement: Supplementary file 6 — Genome wide distribution of iHS and XP-EHH plot. (PDF 349 kb) [file 12864_2018_4759_MOESM6_ESM.pdf]
